# Knowledge, attitudes, and practice towards allergic rhinitis in patients with allergic rhinitis: a cross-sectional study

**DOI:** 10.1186/s12889-023-16607-6

**Published:** 2023-08-25

**Authors:** Wenzhe Gu, Daonan Yan, Zijiang Yuan, Xiaoting Jiang, Yuhan Qian, Hongjun Dong, Zhengjie Shen

**Affiliations:** 1 Department of Otolaryngology, Zhangjiagang Traditional Chinese Medicine Hospital, Affiliated to Nanjing University of Chinese Medicine, Zhangjiagang, 215600 China; 2 Nanjing University of Traditional Chinese Medicine, Nanjing, 210023 China; 3 Department of Oncology, Zhangjiagang Traditional Chinese Medicine Hospital, Affiliated to Nanjing University of Chinese Medicine, Zhangjiagang, 215600 China

**Keywords:** Knowledge, attitudes, practice, Patients, Allergic rhinitis, Cross-sectional study

## Abstract

**Background:**

The knowledge, attitude, and practice (KAP) of Chinese patients with allergic rhinitis (AR) on AR is poorly known. This study investigated the KAP towards AR in patients with this disease and explored the factors associated with KAP.

**Methods:**

This cross-sectional study enrolled patients with AR in Zhangjiagang Hospital of Traditional Chinese Medicine between October 2022 and March 2023.

**Results:**

This study included 656 valid questionnaires. Most participants were 26–35 years old (36.13%) and were female (55.18%). The knowledge, attitude, and practice scores were 5.70 ± 2.88 (possible range: 0–12), 29.51 ± 3.52 (possible range: 9–45), and 34.13 ± 7.55 (possible range: 9–45), indicating poor knowledge, unfavorable attitudes, and proactive practice. AR history of 3–5 years (adjusted odds ratio (adjOR) = 1.62, 95% confidence interval (CI): 1.03–2.54, *P* = 0.037), AR history of > 6 years (adjOR = 1.64, 95%CI: 1.06–2.54, *P* = 0.027), and know their own allergens (adjOR = 2.34, 95%CI: 1.28–4.25, *P* = 0.005) were independently associated with the sufficient knowledge. AR history of ≥ 6 years (adjOR = 0.60, 95%CI: 0.37–0.96, *P* = 0.035), and liking sports (adjOR = 1.58, 95%CI = 1.07–2.33, *P* = 0.020) were independently associated with the positive attitude. The knowledge scores (adjOR = 1.14, 95%CI: 1.05–1.22, *P* = 0.001), attitude scores (adjOR = 1.24, 95%CI: 1.17–1.32, *P* < 0.001), age 36–45 (adjOR = 2.13, 95%CI: 1.19–3.82, *P* = 0.011), employed (adjOR = 0.59, 95%CI: 0.37–0.94, *P* = 0.026), and liking sports (adjOR = 2.11, 95%CI: 1.43–3.14, *P* < 0.001) were independently associated with the proactive practice.

**Conclusions:**

Patients with AR have poor knowledge and unfavorable attitudes but good practice toward AR. Continuous quality teaching interventions and education on patients for AR were recommended.

**Supplementary Information:**

The online version contains supplementary material available at 10.1186/s12889-023-16607-6.

## Background

Allergic rhinitis (AR) is a common type I hypersensitivity response of the upper respiratory tract to seasonal and perennial aeroallergens [1, 2]. Associated conditions include asthma, atopic dermatitis, allergic conjunctivitis, nasal polyps, sinusitis, and sleep apnea [1, 2]. Perennial AR is triggered by dust mites, indoor molds, animal dander, pollen in some climates, and occupational allergens [1]. The risk factors of AR include a family history of atopy, serum IgE > 100 units/mL before age 6 years, higher socioeconomic class, atopy, and air pollution [3]. The prevalence of AR varies widely from 2.2% in Iran to 45.1% in Paraguay, and the prevalence of AR is 18% in China [4, 5]. The management of AR is based on allergen avoidance, intranasal corticosteroids, antihistamines, anticholinergics, nasal saline irrigation, and immunotherapy [2, 6–8]. Poorly controlled symptoms of AR can contribute to sleep loss, secondary daytime fatigue, learning impairment, decreased overall cognitive functioning, decreased long-term productivity, and decreased quality of life, making AR an important public health issue [1].

Knowledge, attitudes, and practice (KAP) surveys allow the identification of misconceptions that could be targeted in the future to improve the subject’s performance [9, 10]. Proper knowledge of AR can allow the patients to adopt adequate behaviors to decrease the attacks and improve their prognosis. The patients’ KAP on AR varies among countries [11–15]. Indeed, an international study showed wide variations among and within countries in perceptions of AR, with significant differences between physicians and patients [11]. A study in Saudi Arabia showed poor KAP of patients with AR toward intranasal corticosteroids [12]. Similar results were observed in rural India [13] and Southeast Asian nations [15]. The KAP of Chinese patients with AR on AR is poorly known.

Therefore, this study aimed to evaluate the KAP towards AR in patients with AR and the factors associated with KAP.

## Methods

### Study design and participants

This cross-sectional study enrolled patients with AR in Zhangjiagang Hospital of Traditional Chinese Medicine, Suzhou, Jiangsu, between October 2022 and March 2023. The inclusion criteria were (1) 18–80 years old, (2) diagnosed with AR, and (3) volunteered to participate in the study and able to understand the questionnaire. The study was approved by the ethics committee of Zhangjiagang Traditional Chinese Medicine Hospital (approval #JS202209021), and informed consent was obtained from the participants.

### Questionnaire

The questionnaire was designed based on previous studies [16, 17] and the Chinese guideline for the diagnosis and treatment of allergic rhinitis (2022 revision) [18]. Forty-one patients were randomly selected for the reliability test, and Cronbach’s α was 0.8622, indicating good internal consistency.

The final questionnaire included demographic data (age, gender, education level, place of residence, type of job, monthly household income, years of illness, allergen test or not, allergen knowledge, and sports), knowledge dimension, attitude dimension, and practice dimension. The knowledge dimension included 12 questions, with 1 point for correct answers and 0 points for incorrect or unclear answers. The score range was 0–12 points. The attitude dimension included 9 questions using a five-point Likert scale. Questions involved in positive attitude were assigned positive values from strongly agree (5 points) to strongly disagree (1 point). Questions involved in negative attitudes (items A1 and A4) were assigned negative values. The score range was 9–45 points. The practice dimension included 9 questions, using a five-point Likert scale, from always (5 points) to never (1 point). The score range was 9–45 points.

Sufficient knowledge, positive attitude, and proactive practice were defined as scores > 70% in each dimension [19].

### Questionnaire distribution and quality control

The e-questionnaires were established using the Sojump platform in China, which generated the QR code. The participants logged in and filled out the questionnaire by scanning the QR code sent by WeChat. In order to ensure the quality and completeness of the questionnaires, only one submission per IP address was allowed, and all items were mandatory. The researchers and trained research assistants assisted in explaining the aim of the study and answered the questions reported by the participants without intervening in the answering process. The researchers examined all questionnaires for completeness, internal coherence, and reasonableness, and questionnaires with repeated options were considered invalid.

### Statistical analysis

SPSS 22.0 (IBM Corp., Armonk, NY, USA) was used for statistical analysis. The continuous variables (i.e., the KAP scores) were presented as means ± standard deviation (SD). The continuous variables were tested for normal distribution. Those with a normal distribution were tested using Student’s t-test (two groups) or ANOVA (more than two groups). Those with a non-normal distribution were tested using the Mann-Whitney U-test (two groups) or Kruskal-Wallis analysis of variance (more than two groups). The demographic data and distribution of knowledge, attitude, and practice dimensions were presented as n (%). Correlations between KAP dimensions were assessed using Pearson’s test. Multiple logistic regression analyses were performed to identify the factors associated with KAP. The variables with *P*-values < 0.05 in univariable analysis were included in the multiple logistic regression analysis. Two-sided *P*-values < 0.05 were considered statistically significant.

## Results

### Characteristics of the participants

In this study, 664 questionnaires were collected, but eight were excluded because all options were repeated. Therefore, 656 valid questionnaires were included in the analysis. As shown in Table 1, most participants were 26–35 (36.13%), female (55.18%), living in urban areas (60.67%), with high school/technical school education or below (54.88%), had long-term stable jobs (50.30%), had a monthly household income of 5000-10,000 (41.01%), had a history of AR of < 3 years (51.22%), were tested for allergens (59.91%), known their allergens (58.54%), and liked sports (54.88%).

**Table 1:** Characteristics of the participants

	n (%)	Knowledge		Attitude		Practice	
		Score	P	Score	P	Score	P
**Total**	656	5.70 ± 2.88		29.51 ± 3.52		34.13 ± 7.55	
**Age**			< 0.001		0.182		0.001
≤ 25	146 (22.26)	6.56 ± 2.38		29.76 ± 3.31		34.81 ± 6.95	
26–35	237 (36.13)	5.97 ± 2.93		29.75 ± 3.67		34.66 ± 7.45	
36–45	153 (23.32)	5.48 ± 2.93		29.30 ± 3.57		34.59 ± 7.90	
≥ 46	120 (18.29)	4.38 ± 2.82		29.00 ± 3.39		31.67 ± 7.61	
**Gender**			0.335		0.836		0.578
Male	294 (44.82)	5.57 ± 2.87		29.54 ± 3.75		33.95 ± 7.98	
Female	362 (55.18)	5.79 ± 2.89		29.48 ± 3.33		34.28 ± 7.19	
**Residence**			< 0.001		0.921		0.001
Rural/suburban	258 (39.33)	5.17 ± 2.92		29.49 ± 3.68		32.96 ± 7.88	
Urban	398 (60.67)	6.04 ± 2.80		29.52 ± 3.42		34.89 ± 7.25	
**Education**			< 0.001		0.347		0.454
High school/technical secondary school and below	360 (54.88)	5.28 ± 2.95		29.39 ± 3.53		33.93 ± 7.75	
College degree or above	296 (45.12)	6.20 ± 2.72		29.65 ± 3.52		34.37 ± 7.32	
**Type of job**			0.107		0.912		0.002
Employed	330 (50.30)	5.52 ± 2.96		29.49 ± 3.78		33.22 ± 7.81	
Unemployed	326 (49.70)	5.88 ± 2.79		29.52 ± 3.25		35.06 ± 7.18	
**Monthly household income, CNY**			0.026		0.550		0.315
< 5000	170 (25.91)	5.53 ± 2.77		29.52 ± 3.47		33.89 ± 7.31	
5000-10,000	269 (41.01)	5.48 ± 2.94		29.70 ± 3.51		33.94 ± 7.91	
10,001–20,000	151 (23.02)	5.87 ± 2.95		29.19 ± 3.49		34.01 ± 7.53	
> 20,000	66 (10.06)	6.61 ± 2.58		29.44 ± 3.80		35.79 ± 6.66	
**Duration of disease**			< 0.001		0.139		0.161
< 3 years	336 (51.22)	4.92 ± 3.02		29.76 ± 3.67		33.61 ± 8.46	
3–5 years	146 (22.26)	6.32 ± 2.55		29.36 ± 3.21		34.97 ± 6.49	
≥ 6 years	174 (26.52)	6.67 ± 2.43		29.14 ± 3.48		34.43 ± 6.41	
**Allergens test**			< 0.001		0.191		< 0.001
Yes	393 (59.91)	6.58 ± 2.26		29.66 ± 3.19		35.59 ± 6.46	
No	263 (40.09)	4.37 ± 3.18		29.29 ± 3.96		31.95 ± 8.50	
**Known your allergens**			< 0.001		0.204		< 0.001
Yes	384 (58.54)	6.71 ± 2.22		29.66 ± 3.26		35.57 ± 6.45	
No	272 (41.46)	4.26 ± 3.09		29.30 ± 3.86		32.10 ± 8.49	
**Like sports**			0.002		0.004		< 0.001
Yes	360 (54.88)	6.02 ± 2.70		29.87 ± 3.52		35.79 ± 7.05	
No	296 (45.12)	5.30 ± 3.04		29.07 ± 3.49		32.11 ± 7.67	

### Knowledge

The mean knowledge score was 5.70 ± 2.88 (possible range: 0–12), indicating poor knowledge. Higher knowledge scores were observed in younger participants (*P* < 0.001), urban residency (*P* < 0.001), higher education (*P* < 0.001), with higher income (*P* = 0.026), with longer AR duration (*P* < 0.001), passed allergen tests (*P* < 0.001), known allergens (*P* < 0.001), and liking sports (*P* = 0.002) (Table 1). Poor knowledge was observed for specific knowledge items, including K2 (65.70%; “The allergens that cause rhinitis vary depending on where you live”), K4 (46.80%; “Allergic rhinitis is often accompanied by asthma”), K5 (4.12%; “Skin prick test, as one kind of allergen test, lead to no side effects”), K6 (42.68%; “Blood tests are more objective than skin prick test”), K7 (53.96%; “Allergic rhinitis is partly hereditary”), K8 (49.09%; “Allergic rhinitis requires long-term medication”), K9 (64.33%; “Sublingual desensitization is a treatment for allergic rhinitis”), K10 (45.43%; “Chinese medicine does not affect treating allergic rhinitis”), K11 (9.76%; “Nasal glucocorticoids (topical) bring the most significant efficacy for allergic rhinitis compared to other treatments”), and K12 (10.37%; “Surgery can cure allergic rhinitis”) (Supplementary Table S1).

### Attitudes

The mean attitude score was 29.51 ± 3.52 (possible range: 9–45), indicating unfavorable attitudes. Higher attitude scores were observed in participants who liked sports (*P* = 0.004) (Table 1). The distribution of the attitude levels is shown in Supplementary Figure S1.

### Practice

The mean practice score was 34.13 ± 7.55 (possible range: 9–45), indicating good practice. Higher attitude scores were observed in younger participants (*P* = 0.001), urban residents (*P* = 0.001), with non-long-term stable jobs (*P* = 0.002), passed allergen tests (*P* < 0.001), known allergens (*P* < 0.001), and liking sports (*P* < 0.001) (Table 1). The distribution of the practice levels is shown in Supplementary Figure S2.

### Correlations

The knowledge scores are correlated with the attitude (*r* = 0.120, *P* = 0.002) and practice (*r* = 0.378, *P* < 0.001) scores. The attitude scores were correlated with the practice scores (*r* = 0.398, *P* < 0.001) (Table 2).

**Table 2:** Correlation analysis

	Knowledge	Attitude	Practice
**Knowledge**	1		
**Attitude**	0.120 (*P* = 0.002)	1	
**Practice**	0.378 (*P* < 0.001)	0.398 (*P* < 0.001)	1

### Multiple logistic regression analysis

AR history of 3–5 years (adjOR = 1.62, 95%CI: 1.03–2.54, *P* = 0.037), AR history of ≥ 6 years (adjOR = 1.64, 95%CI: 1.06–2.54, *P* = 0.027), and know their own allergens (adjOR = 2.34, 95%CI: 1.28–4.25, *P* = 0.005) were independently associated with the sufficient knowledge (Table 3).

**Table 3:** Multiple logistic regression analysis

	Knowledge		Attitude		Practice	
	adjOR (95%CI)	P	adjOR (95%CI)	P	adjOR (95%CI)	P
**Knowledge**			1.01 (0.94 1.09)	0.771	1.14 (1.05 1.22)	0.001
**Attitude**					1.24 (1.17 1.32)	< 0.001
**Age**						
≤ 25	Ref.		Ref.		Ref.	
26–35	1.36 (0.84 2.20)	0.217	1.04 (0.63 1.72)	0.881	1.27 (0.76 2.11)	0.361
36–45	1.02 (0.58 1.78)	0.958	0.63 (0.35 1.15)	0.130	2.13 (1.19 3.82)	0.011
≥ 46	0.62 (0.32 1.23)	0.173	0.58 (0.29 1.13)	0.110	0.69 (0.34 1.39)	0.299
**Gender**						
Male	Ref.		Ref.		Ref.	
Female	1.33 (0.92 1.92)	0.133	0.95 (0.65 1.39)	0.792	1.24 (0.84 1.82)	0.281
**Residence**						
Rural/suburban	Ref.		Ref.		Ref.	
Urban	1.19 (0.81 1.75)	0.382	0.81 (0.55 1.19)	0.281	1.30 (0.86 1.94)	0.209
**Education**						
High school/technical secondary school and below	Ref.		Ref.		Ref.	
College degree or above	1.39 (0.89 2.19)	0.151	1.16 (0.73 1.85)	0.531	1.15 (0.71 1.86)	0.563
**Type of job**						
Employed	0.99 (0.64 1.54)	0.965	1.21 (0.77 1.89)	0.407	0.59 (0.37 0.94)	0.026
Unemployed	Ref.		Ref.		Ref.	
**Monthly household income, CNY**						
< 5000	Ref.		Ref.		Ref.	
5000-10,000	1.01 (0.63 1.59)	0.983	0.83 (0.52 1.30)	0.408	1.34 (0.83 2.16)	0.231
10,001–20,000	1.31 (0.77 2.22)	0.315	0.65 (0.38 1.13)	0.125	1.68 (0.96 2.93)	0.069
> 20,000	1.17 (0.60 2.26)	0.648	0.71 (0.34 1.47)	0.358	1.81 (0.91 3.61)	0.093
**Duration of disease**						
< 3 years	Ref.		Ref.		Ref.	
3–5 years	1.62 (1.03 2.54)	0.037	0.67 (0.41 1.09)	0.107	0.70 (0.43 1.14)	0.150
≥ 6 years	1.64 (1.06 2.54)	0.027	0.60 (0.37 0.96)	0.035	0.75 (0.47 1.21)	0.239
**Allergens test**						
Yes	1.02 (0.56 1.86)	0.947	0.87 (0.48 1.59)	0.655	1.21 (0.64 2.29)	0.563
No	Ref.		Ref.		Ref.	
**Known your allergens**						
Yes	2.34 (1.28 4.25)	0.005	1.09 (0.60 1.99)	0.779	1.33 (0.71 2.49)	0.378
No	Ref.		Ref.		Ref.	
**Like sports**						
Yes	1.39 (0.96 2.02)	0.085	1.58 (1.07 2.33)	0.020	2.11 (1.43 3.14)	< 0.001
No	Ref.		Ref.		Ref.	

*adjOR* Adjusted odds ratio, *CI* Confidence interval

AR history of ≥ 6 years (adjOR = 0.60, 95%CI: 0.37–0.96, *P* = 0.035) and liking sports (adjOR = 1.58, 95%CI = 1.07–2.33, *P* = 0.020) were independently associated with the positive attitude (Table 3).

The knowledge scores (adjOR = 1.14, 95%CI: 1.05–1.22, *P* = 0.001), attitude scores (adjOR = 1.24, 95%CI: 1.17–1.32, *P* < 0.001), age 36–45 (adjOR = 2.13, 95%CI: 1.19–3.82, *P* = 0.011), employed (adjOR = 0.59, 95%CI: 0.37–0.94, *P* = 0.026), and liking sports (adjOR = 2.11, 95%CI: 1.43–3.14, *P* < 0.001) were independently associated with the proactive practice (Table 3).

## Discussion

The results showed that patients with AR in Suzhou, Jiangsu, have poor knowledge and unfavorable attitudes but good practices toward AR. Teaching interventions should be designed to improve knowledge levels. Since the management of AR involves self-management, behaviors, and life habits, better knowledge and attitudes toward AR should translate into better clinical outcomes among patients with AR.

Besides medication, managing AR involves avoiding allergens and having good life habits [2, 6–8]. Therefore, a high level of KAP plays a major role in the management of AR. The present study revealed poor knowledge and unfavorable attitudes of patients with AR towards AR but active practice. The results also suggested that the knowledge and attitude scores were independently associated with the practice scores, but the correlations were weak. Therefore, the results suggest that even though knowledge and attitudes participate in practice levels, several patients pose actions out of habit or following advice without understanding why they perform those actions. Interestingly, the multinational study by Bhargave et al. [11] revealed important discrepancies among countries regarding the KAP of patients and physicians toward AR. The KAP of patients with AR in Saudi Arabia was reported to be low [12, 14]. Similar results were reported in India [13] and four Southeast Asian nations [15]. Thai patients have poor knowledge of the risks of immunotherapy for AR [20], and similar results were observed in German athletes with AR [21]. A population study in Nigeria showed that 16% of the participants had knowledge of AR symptoms, and 11% had AR [22, 23]. These studies generally support the present study. Indeed, among the knowledge items, only two showed good knowledge: symptoms of AR and dust mites as a cause of AR. All other knowledge items had poor results, with the poorest being the possible side effects of the skin prick test, the efficacy of glucocorticoids, and the use of surgery to cure AR. Previous KAP studies on AR also reported a wide array of misconceptions about the management of AR [11, 12, 14, 24]. The misconceptions mainly regarded the use of corticosteroids and smoking [12], the causative agents [14], AR manifestations [22–24], and risks of immunotherapy [20, 21]. Previous studies generally support the present one, with variable but generally low/moderate KAP [11–15, 20–22]. Differences among studies can be due to the general health literacy of a population, education, and the healthcare system, as well as the tools designed to assess KAP.

The present study in Suzhou showed that a longer course of AR and knowing the culprit allergens were associated with a better knowledge of AR. A longer course of AR, having passed allergen tests, and liking sports were associated with better attitudes. Liking sports and having a non-stable job were associated with better practice. These results could help identify the patients with a higher need for KAP interventions. Alreshidi et al. [14] reported that only a higher education degree was associated with higher KAP, which was also observed in the present study but only in the univariable analysis. Al-Rasheedi [12] showed that AR knowledge was associated with education, follow-up facilities, and smoking. Of course, differences among studies can be due to the variables selected. In addition, the results of KAP studies are only applicable to the specific population that was surveyed, and subsequent recommendations can only be applied to the surveyed population, i.e., Suzhou, for the present study. Of note, the smoking status was not collected in the present study. Smoking is a risk factor for AR and can reduce the effectiveness of corticosteroids [25, 26]. Hence, smoking cessation should be encouraged. Since education can be variable among the patients with AR, care should be taken to deliver information on AR that is understandable to all patients [27, 28]. Lee et al. [28] highlighted that patients must understand the instructions correctly to receive optimal corticosteroid treatments. Participants with an AR history of > 6 years were less likely to have a positive attitude toward AR compared to those with a shorter AR history. In addition, participants who like sports are more likely to have a positive attitude compared with those who do not. Being employed was associated with a lower likelihood of engaging in proactive practices toward AR compared to those who are not employed, probably because of time constraints in everyday life.

The present study showed positive correlations among knowledge, attitudes, and practices, as observed in previous studies [12, 29], indicating that improving knowledge should translate into better attitudes and practices toward AR. Hence, education interventions should be designed and implemented to improve the KAP of patients with AR. Community healthcare professionals in direct contact with the patients are often the main source of medical information, and a study suggested that community pharmacists should actively participate in the KAP of patients with AR by suggesting proper treatments [12].

This study had limitations. The participants were from a single hospital deserving a limited area in China, limiting the generalizability of the results, especially in the context where the allergens can vary with geography and climate. Considering the prevalence of AR, the sample size was small. As for all cross-sectional KAP surveys, the data represent a specific point in time [9, 10]. No previous data were available for comparison. Another limitation inherent to all KAP surveys is the social desirability bias, in which the participants can be tempted to answer the socially correct answer instead of answering what they do [30, 31]. Validity could not be tested on the pretest sample due to the too-small sample size. Nevertheless, validity was evaluated in the whole study population (*n* = 656), and the KMO was 0.894. Finally, the correlations between KAP dimensions were weak, but statistically significant, indicating that there not due to chance alone. Still, it suggests that several factors are acting together to influence the KAP dimensions.

In conclusion, patients with AR in the Suzhou area have poor knowledge and unfavorable attitudes but good practices toward AR. Continuous quality teaching interventions and education on patients for AR were recommended.

### Supplementary Information

**Additional file 1: Supplementary Table S1.** Distribution of knowledge dimension.

**Additional file 2: Supplementary Figure S1.** Distribution of the attitude dimension. **Supplementary Figure S2.** Distribution of the practice dimension.

## Data Availability

All data generated or analyzed during this study are included in this published article.

## Abbreviations

KAP: Knowledge, attitude, and practice
AR: Allergic rhinitis
