# Supplementary material for: Knowledge, attitudes, and practice towards allergic rhinitis in patients with allergic rhinitis: a cross-sectional study
Source: BMC Public Health. 2023 Aug 25;23:1633. doi: 10.1186/s12889-023-16607-6 (PMC10464446; doi:10.1186/s12889-023-16607-6)
Supplement: Supplementary file 1 — Additional file 1: Supplementary Table S1. Distribution of knowledge dimension. [file 12889_2023_16607_MOESM1_ESM.docx]

**Supplementary Table S1.** Distribution of knowledge dimension

| Knowledge | Accuracy, n (%) |
| --- | --- |
| 1. The typical symptoms of allergic rhinitis are paroxysmal sneezing, watery nose, nasal itching, and nasal congestion, which may be accompanied by ocular symptoms. | 535 (81.55) |
| 2. The allergens that cause rhinitis vary depending on where you live. | 431 (65.70) |
| 3. Dust mites are common allergens of allergic rhinitis. | 511 (77.90) |
| 4. Allergic rhinitis is often accompanied by asthma. | 307 (46.80) |
| 5. Skin prick test, as one kind of allergen test, lead to no side effects. | 27 (4.12) |
| 6. Blood tests are more objective than skin prick tests. | 280 (42.68) |
| 7. Allergic rhinitis is partly hereditary. | 354 (53.96) |
| 8. Allergic rhinitis requires long-term medication. | 322 (49.09) |
| 9. Sublingual desensitization is a treatment for allergic rhinitis. | 422 (64.33) |
| 10. Chinese medicine has no effect on treating allergic rhinitis. | 298 (45.43) |
| 11. Nasal glucocorticoids (topical) bring the most significant efficacy for allergic rhinitis compared to other treatments. | 64 (9.76) |
| 1. Surgery can cure allergic rhinitis. | 68 (10.37) |
