# Supplementary material for: Knowledge, attitudes, and practice towards allergic rhinitis in patients with allergic rhinitis: a cross-sectional study
Source: BMC Public Health. 2023 Aug 25;23:1633. doi: 10.1186/s12889-023-16607-6 (PMC10464446; doi:10.1186/s12889-023-16607-6)
Supplement: Supplementary file 2 — Additional file 2: Supplementary Figure S1. Distribution of the attitude dimension. Supplementary Figure S2. Distribution of the practice dimension. [file 12889_2023_16607_MOESM2_ESM.docx]

**
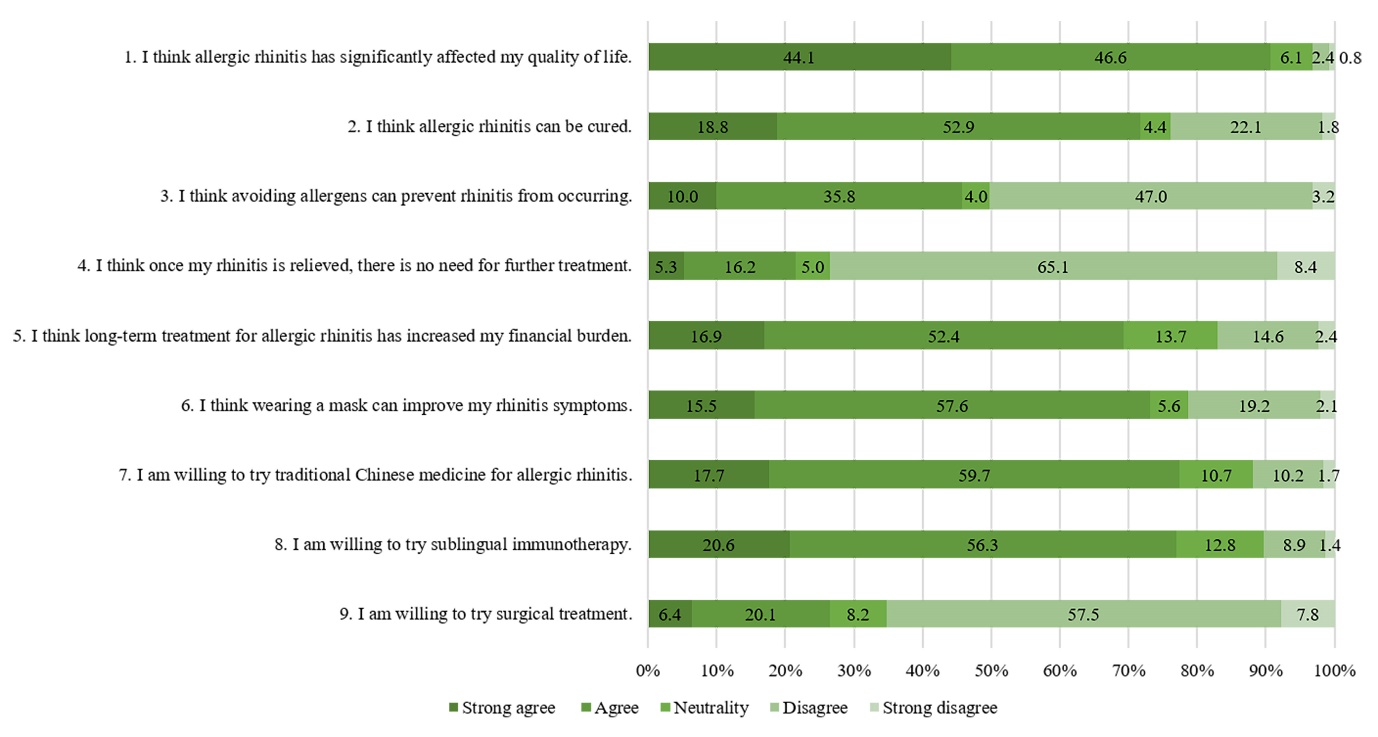
**

**Supplementary Figure S1.** Distribution of the attitude dimension

**
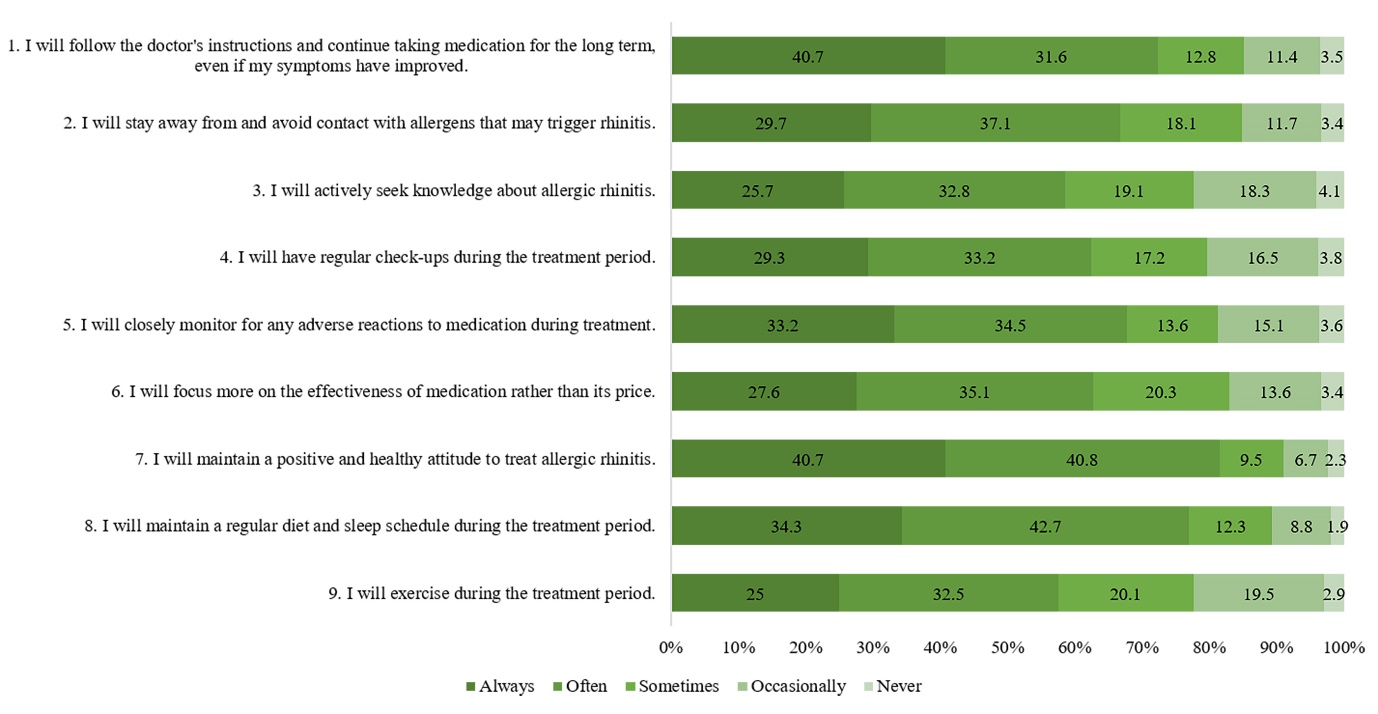
**

**Supplementary Figure S2.** Distribution of the practice dimension.
